# Communication to Pediatric Cancer Patients and their Families: A Cultural Perspective

**DOI:** 10.4103/0973-1075.63131

**Published:** 2010

**Authors:** Tulika Seth

**Affiliations:** Assistant Professor in Hematology, Room 5017, Teaching Block, AIIMS, New Delhi, India

**Keywords:** Counseling, Cultural acceptable, Need based stress management

## Abstract

**Background:**

Communication is a key component of palliative care. The area of pediatric palliative care is emotionally distressing for families and healthcare providers. Inadequate communication can increase the stress and lead to mistrust or miscommunication.

**Materials and Methods:**

Reviewing the literature on communication between physicians and patients, we identified several barriers to communication such as paternalism in medicine, inadequate training in communication skills, knowledge of the grieving process, special issues related to care of children and cultural barriers. In order to fill the void in area of cultural communication, a study questionnaire was administered to consecutive families of children receiving chemotherapy at a large, north Indian referral hospital to elicit parental views on communication.

**Results:**

Most parents had a protective attitude and favored collusion, however, appreciated truthfulness in prognostication and counseling by physicians; though parents expressed dissatisfaction on timing and lack of prior information by counseling team.

**Conclusion:**

Training programs in communication skills should teach doctors how to elicit patients’ preferences for information. Systematic training programs with feedback can decrease physicians stress and burnout. More research for understanding a culturally appropriate communication framework is needed.

## INTRODUCTION

Communication is a very important part of palliative care. Effective communication is clear and unambiguous, it is required to inform the patient about the disease, treatment, prognosis, course of illness and complications; in case of a terminal disease –what to expect, options and time left. This communication helps to allay fears of the unknown and provides empowering information. It ensures that the patient and the treating and/or palliative care team have a clear understanding of the goals and course of action. As the patient and family require repeated reassurance, this communication has become synonymous with counseling. A term which aptly describes this inter action is ‘collaborative communication,’ coined by Feundtner.[1] In most Indian cancer centers the physicians undertake this communication /counseling themselves; a few centers also have psychologists to provide additional counseling. Current available guidelines for communication are based on needs and wishes of western patients.

### Problems caused by inadequate communication

Without proper communication the patients and their families will find it hard to make sense of the grave situation. In their distress they will search for information from various sources, often ending up with information which is incorrect or not applicable to their case or not congruent with the treatment plan. Because of these problems, they may find it difficult to maneuver through the complex maze of cancer therapy. This can result in abandonment of treatment or refusal of various curative modalities due to increased perceived risk or lack of perceived benefit. When things go badly and the disease is unresponsive to treatment or has relapsed, communication channels are further strained.

### Important issues to consider in pediatric palliative care communication

#### Paternalism in medicine

Patient autonomy has become widely popular and given the traditional paternalistic style of medicine a negative connotation. Recent literature has focused on patient and families rights and the need to give due importance to their wishes in treatment decision making. However, many patients themselves may want the traditional physician’s role of the sole decision maker, as described by Rodriguez-Osorio and Dominguez-Cherit.[2] A middle path is required, the patient and families should be allowed to discuss and determine the course, as long as this is an informed decision and they are aware of the alternatives, risks and benefits.

#### Training and skills in communication

Without systematic training, breaking of bad news and discussions of cancer prognosis will fall short of existing guidelines and patients’ needs and expectations. Consultations of this type can be difficult and painful. Many doctors lack counseling skills and frequently delegate the important task of giving bad news to their resident, the palliative care team or may superficially discuss recurrence of disease without imparting details of what this means to the patient. Use of euphemisms, for relapse and death are common. This can lead to families often being unaware of the gravity of the situation, as they cling to hope, until the truth dawns on them. This inadequate communication can lead to distress, disappointment and anger towards the team, despite the care being provided.

A study amongst Clinical Oncology faculty showed that as few as six per cent had received formal training in delivering bad news. Many faculty members rated their own skills to communicate bad news as inadequate. The randomized clinical trial of communication skills training with facetoface learning with patients or simulated patient, and feedback by Arnold and Koczwara,[3] has provided research evidence of benefit of this technique.

Inadequate physician-patient communication skills may lead to psychological distress, increased anxiety and poor psychological adjustment to cancer which has been elucidated by Liénard *et al*.[4] Reducing the level of patients’ anxiety is important because anxiety may lead to emotional distress and functioning disturbances as found by Arnold and Koczwara[3] and Liénard *et al*;[4] this may interfere with patients’ compliance as found by Girgis and Sanson-Fisher.[5] A study by Liénard *et al*,[4] on the effect of training, however, did not show any measurable impact of training the reasons for this finding are perhaps because counseling is an inherent skill, or that this skill is not well assessed by current research tools. However, practice, and following guidelines for developing expertise in communication and receiving feedback from mentors does make the task more structured and less stressful to the physician or healthcare provider as shown by the work of Girgis and Sanson-Fisher[5] and Maguire.[6,7]

#### Knowledge of counseling

The Kübler-Ross model, also known as the five stages of grief, is a concept that is familiar to most. It states that the process of dealing with a terminal illness or news of cancer has five discrete stages - denial, anger, bargaining, depression and acceptance. This has created awareness and understanding about the grieving process. However, all people do not go through the process in sequence, or reach to acceptance. Often people experience several stages in a “roller coaster” effect - switching between two or more stages, returning to one several times before being able to move on, as described by the pioneering work of Kübler-Ross.[8] The limitation of the Kübler-Ross model is that it neglects the effects of other stressors which act as an impediment to going through the normal grieving process e.g. lack of support, financial problems, the effect of illness on the person, which were high- lighted by Kastenbaum.[9]

Going through the process takes time and communication needs to be strong, all members of the team should reenforce the same information as the family will not be able to comprehend much during this stressful period. Patience is needed to repeat information and ensure correct processing, and avoid confusion.

#### Pediatric palliative care

Most physicians feel a burden to cure the patient, and failure of therapy is often considered a failure by the patient to respond to treatment or a personal failure by the doctor. Caring for children is a very emotive experience, to see a child suffering is sad, and a child with a terminal illness is especially difficult, as it is a life cruelly shortened and unfulfilled. Many health providers will identify patients with their own children of the same age, or are hampered by feelings of personal sorrow, and find it difficult to communicate appropriately with the families. Communication often focuses only on the next treatment to be done, and emotional or distressing facts are not discussed. Healthcare Providers inhibitions, are a major hurdle to effective communication. Healthcare providers may need to seek counseling themselves or at least discuss their issues with colleagues to prevent burnout as explored by Whippen and Canellos.[10]

Many teenagers and young adults are kept in the dark by their families and palliative care team to avoid distress and fear. Fear of the unknown can be a greater stress factor; loss of autonomy can frustrate the child. Not talking to the child puts up barriers to communication, making the child afraid to ask questions about death or suffering. Further, the child may be deprived of counseling to alleviate the distressful emotions.

#### Cultural communication

In India there are many socio- economic and cultural issues that can become barriers for treatment. There are parents who have reservations about western medicine, feeling they are too strong or too hot for children. Many families use complementary medicines as well, but are fearful of informing the treating doctors. The parents are often unwilling to undertake any type of major surgery or treatment that will compromise on the female child’s physical appearance or lead to permanent disability e.g. deformity, loss of vision; as it will hamper future prospects of marriage. If therapy is likely to cause infertility there are parental concerns about such treatments, as they fear societal stigma for the child when she grows up.

Western concepts of telling the cancer patient about the disease and involving the patient/or family in treatment making decisions are alien to the Indian culture and may add to the patients distress. Collusion to keep the patient unaware of the diagnosis is well described by Chaturvedi, Loiselle and Chandra.[11] The family often believes that telling the truth will rob the patient of hope, as elucidated in Moore and Butow.[12]

### Research needs

Palliative care in India is developing rapidly and many articles on pain and symptom control are available, and cultural counseling guidelines are available from many other countries, such as the detailed book by Moore and Spiegel.[13] This area needs research from many view points, as a step forward we conducted a pilot study at our hospital to define the current, cultural milieu and provide a framework in which healthcare providers can apply established techniques of counseling and provide communication that is needed, sensitive, and acceptable.

### Aims

1. To explore parental perceptions to telling a child suffering from cancer about his/her disease, prognosis and treatment in an Indian tertiary care hospital.
2. Assess parental views on participation of children in decision making for treatment, and beliefs and practices for palliative care.

## MATERIALS AND METHODS

After informed consent, 25 consecutive parents of pediatric cancer patients in the age group 10-18 years (mean age 13 years) were interviewed. All the children were on treatment for their disease at our hospital for at least six months (range 6 month to 38 months), all children were suffering from acute lymphoblastic leukemia. They were administered a simple questionnaire which had been formulated using WHO guidelines, and prior tested in the field for clarity and information. Parents were interviewed together, without the child being present.

## RESULTS

All parents who were approached consented to the study. Two families did not complete the study; as the child died in one case and in the other, the family left for alternative medical treatment, these two families refused to complete the interview and per their request their data was excluded from the study, hence 23 interviews were completed and analyzed. Majority of the families had not wanted their children (even aged 18 years) to be informed of the diagnosis [65%, (15/23)], though it was encouraging to see at least 35% had been open to informing the child about their diagnosis. Though almost all [95%, (22/23)] felt the child should not make any decision about the treatment. Majority [60%, (14/23)] felt the child should not even be informed about side effects of therapy and especially about long term effects.

If the diagnosis, prognosis and other information was to be told to the child, 100% preferred that the doctors to give the information to the child. Though they all approved of the amount and content of information given to the child, only 21% (5/23) were satisfied with the timing or manner it was delivered. The parental reservations were that they had not been given sufficient prior knowledge of what was to be done (counseling) and would have liked additional time to prepare themselves and would have wanted the information to have been given to the child at a later date (after treatment started and not before as done by physicians).

Palliative care was a difficult concept to explain and only three families were receiving palliative care at the time of administration of the questionnaire. In these families both child and parent were aware of the prognosis, but the parents felt that they would have liked to have shielded their child from the knowledge if possible. Deciding when to stop curative treatment, when such treatment was futile, was a hypothetical question that was posed to the families. The parental responses showed that the decision was mostly the domain of the parents -20 felt only parents should decide, seven doctors alone and four families stated that both the doctor and parent should decide, none of the parents felt the child should take part in this decision making process.

## DISCUSSION

The study reinforces the already observed parental belief in the traditional paternalistic role of the physician. These families were not under acute psychological stress but in some cases, even with greater than three years of therapy they were still finding it hard to communicate about cancer to their children. Parents of Indian children with cancer are very unwilling to have the news broken to the child and tend to delay the process as much as possible. They do not wish to involve the child in any deliberations for treatment or palliation. This is unlike the west, where the autonomy of a child is given importance and a teenager is given the right to assent or refuse therapy. Indian parents wanted to shield and protect their children even from the knowledge of cancer, as shown by their desire to delay the child’s counseling to after treatment starts and avoid unpleasant discussions on prognosis and side effects, this is an important cultural response, and the physicians need to be aware of it. The treating team needs to forge an alliance with the family to facilitate communication and give the parents time to cope with their own fear and anxiety.

### Limitations of the study

It did not take the individual parent (mother/father) views, but decisions are usually taken by the parents together in Indian families and the dominant view is what realistically happens, hence we felt it gave the actual situation. We did not study the child’s viewpoint in this pilot. Counseling needs are largely unmet and many more studies to provide communication guidelines are required. Better communication with the family to elicit how they would like to receive information will be a useful and important tool for physicians and counselors.

### Future directions

It is important for healthcare providers to give information in clear and simple language and explain things according to the needs of the patient and if the patient is a minor, as *per* the parents preferences. A middle path is required in breaking news. First information is to be given to the family, then gradual discussion of the disease and options with the patient, while providing hope, whenever the patient is ready for communication. In terminal cases the hope is for support, comfort and relief of pain. The training programs in communication skills should also teach doctors how to elicit patients’ preferences for information about information. Despite workshops being effective in changing key communication behaviors, it is not certain how much of what is learnt is applied to clinical practice as pointed out by Maguire.[6,7] The onus is on the healthcare provider to provide effective communication that is required.
